# Age is the work of art? Impact of neutrophil and organism age on neutrophil extracellular trap formation

**DOI:** 10.1007/s00441-017-2751-4

**Published:** 2017-12-18

**Authors:** Weronika Ortmann, Elzbieta Kolaczkowska

**Affiliations:** Department of Evolutionary Immunology, Institute of Zoology and Biomedical Research, Jagiellonian University, ul. Gronostajowa 9, 30-387 Krakow, Poland

**Keywords:** Neutrophil extracellular traps, Immature neutrophils, Mature neutrophils, Neonates, Elderly, Scenescent

## Abstract

Neutrophil extracellular traps or NETs are released by highly activated neutrophils in response to infectious agents, sterile inflammation, autoimmune stimuli and cancer. In the cells, the nuclear envelop disintegrates and decondensation of chromatin occurs that depends on peptidylarginine deiminase 4 (PAD4) and neutrophil elastase (NE). Subsequently, proteins from neutrophil granules (e.g., NE, lactoferrin and myeloperoxidase) and the nucleus (histones) bind to decondensed DNA and the whole structure is ejected from the cell. The DNA decorated with potent antimicrobials and proteases can act to contain dissemination of infection and in sterile inflammation NETs were shown to degrade cytokines and chemokines via serine proteases. On the other hand, overproduction of NETs, or their inadequate removal and prolonged presence in vasculature or tissues, can lead to bystander damage or even initiation of diseases. Considering the pros and cons of NET formation, it is of relevance if the stage of neutrophil maturation (immature, mature and senescent cells) affects the capacity to produce NETs as the cells of different age-related phenotypes dominate in given (pathological) conditions. Moreover, the immune system of neonates and elderly individuals is weaker than in adulthood. Is the same pattern followed when it comes to NETs? The overall importance of individual and neutrophil age on the capacity to release NETs is reviewed in detail and the significance of these facts is discussed.

## Introduction

Neutrophils, polymorphonuclear cells (PMNs), are the first leukocytes to reach the site of inflammation where they perform their effector functions, phagocytose microbes and kill them intracellularly. Alternatively, neutrophils fight pathogens extracellularly in either of two ways: upon discharge of potent antimicrobials and proteases from their granules or on release of neutrophil extracellular traps (NETs; Kolaczkowska and Kubes 2013).

**Fig. 1:**
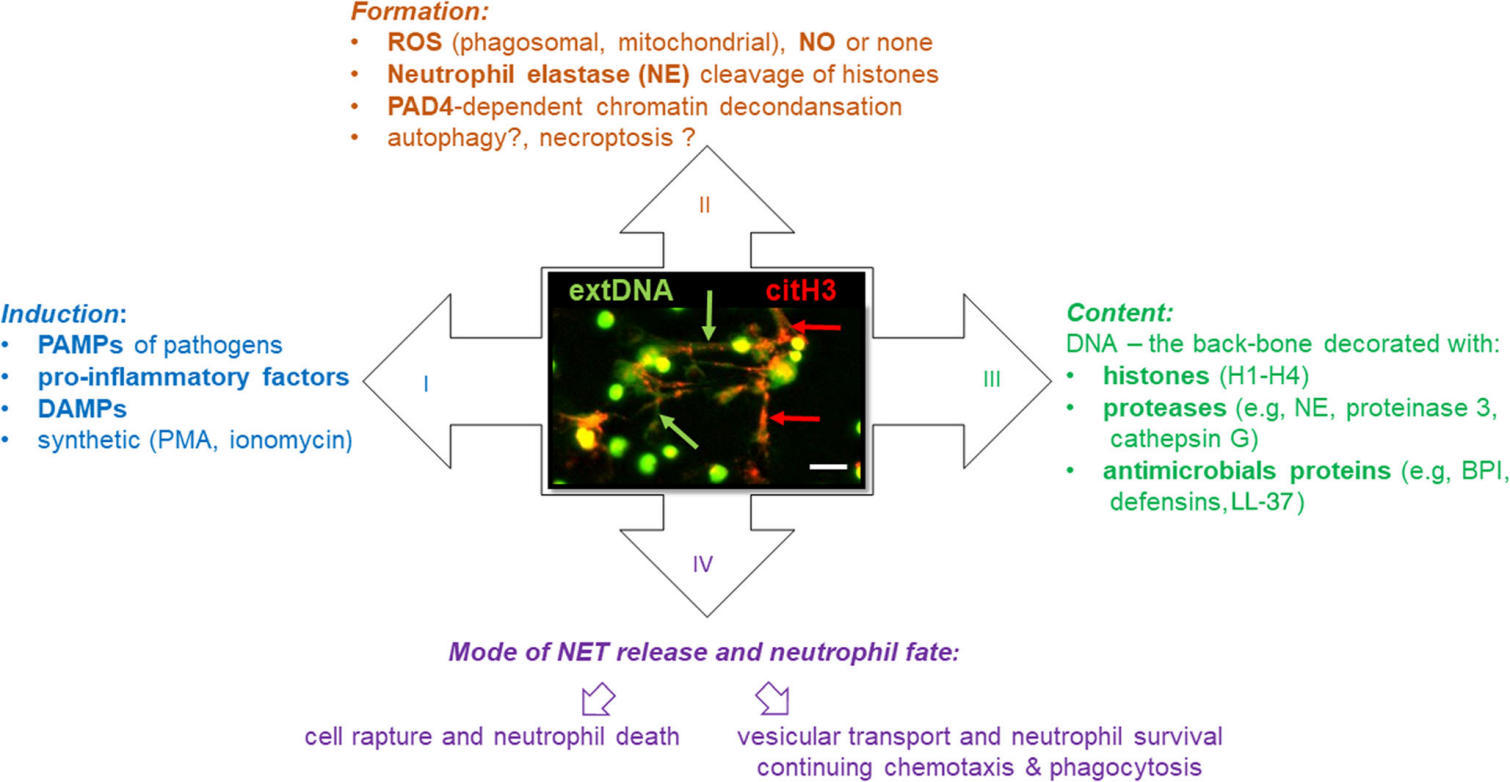
Basic characteristics of neutrophil extracellular traps (NETs): I) inducing factors, II) involved pathways, III) composition and IV) fate of NET-releasing neutrophils. The image captures NETs formed upon LPS stimulation of murine neutrophils (*green arrows* extracellular DNA,* red arrows* citrullinated histone H3),* scale bar* 50 μm.* PAMP* pathogen-associated molecular pattern,* DAMP* damage-associated molecular pattern,* PMA* phorbol 12-myristate 13-acetate,* ROS* reactive oxygen species,  *NO* nitric oxide,* PAD4* peptidylarginine deiminase 4, *MPO* myeloperoxidase, *BPI* bactericidal/permeability-increasing protein,* LL-37* cathelicidins cathelicidin

The first report on NETs revealed that neutrophils stimulated by agents such as lipopolysaccharide (LPS), interleukin 8 (IL-8) or phorbol 12-myristate 13-acetate (PMA) form and release structures similar to the network, hence their name (Brinkmann et al. 2004). Detailed studies of NETs by electron scanning and confocal microscopy as well as proteomic analyses showed that NETs are composed of thin chromatin fibers that are decorated with some 30 neutrophil proteins, including neutrophil elastase (NE), bactericidal/permeability-increasing protein (BPI), defensins, cathelicidin (LL-37), proteinase 3 and cathepsin G of granular origin and nuclear histones (Brinkmann et al. 2004; Urban et al. 2009) (Fig. 1). NETs can take different forms, from a band form, by a cloud-like structure, when the NET is fully hydrated, to a network-like shape, exceeding 10–15 times the volume of the releasing cell (Brinkmann et al. 2004; Brinkmann and Zychlinsky 2012). More recent studies, applying atomic force microscopy to reveal their nanoscale properties, reported that NETs are branching filament networks with a substantially organized porous structure and with openings in the size range of small pathogens (Pires et al. 2016). Importantly, proteases attached to NETs secure assembly of the whole structure and its mechanical properties. While such a structure increases the efficiency of catching pathogens, it can also favor collateral damage (Pires et al. 2016). The latter observation directly relates to pros and cons of NET formation.

## Ying and yang of NETs

There are multiple reports on NETs being able to capture, immobilize and neutralize pathogens. The microbes caught by NET include both Gram-positive (e.g., *Staphylococcus aureus*) and Gram-negative bacteria (e.g., *Salmonella typhimurium* and *Shigella flexneri*; Brinkmann et al. 2004), fungi (e.g., *Candida albicans*; Urban et al. 2006) and viruses (Saitoh et al. 2012; Jenne et al. 2013). More controversial is their capacity to kill trapped pathogens. As NETs are decorated with antimicrobial proteins and proteases, their killing potential seemed to be unavoidable and in fact it was repeatedly reported to occur (Brinkmann et al. 2004; Urban et al. 2006; Guimarães-Costa et al. 2009). However, some studies ruled it out (Gabriel et al. 2010). A recent paper by Menegazzi et al. (2012) challenged the technical approach applied in the majority of the studies, most of which were performed on isolated neutrophils and revealed that the results depended on the chosen strategy; i.e., incubation with DNase prior or post-addition of bacteria to the NET forming neutrophils. Overall, the study concluded that NETs entrap but do not kill microbes (Menegazzi et al. 2012). This is in line with some in vivo studies showing that, after intravascular application of DNase, colony-forming units (CFUs) of *S. aureus* do not increase despite strong deposition of NETs in the vasculature of mice with *S. aureus* sepsis (Kolaczkowska et al. 2015). But even if NETs indeed do not kill pathogens, their role in capturing and immobilizing microbes should not be underestimated as NETs prevent microbial dissemination throughout the body. This was, for example, shown in the course of *Escherichia coli* sepsis (McDonald et al. 2012). Moreover, one can speculate that NETs can indirectly contribute to pathogen killing, as immobilized microbes are exposed to microenvironmental immune factors present in serum or tissues as well as cytotoxic leukocytes (macrophages and NK cells). In addition, by means of proteases attached to NETs, virulence factors of pathogens can be shed from their surface limiting their virulency, e.g., IpaB on *S. flexneri* is being removed by NE decorating the traps (Brinkmann et al. 2004). Another important, anti-inflammatory function of NETs comes from studies on sterile inflammation, as during gout, serine proteases attached to NETs were shown to degrade pro-inflammatory cytokines and chemokines contributing to the resolution of the immune response (Schauer et al. 2014).

The importance of NETs is further strengthened by four facts: (1) their evolutionary conservation, (2) release by multiple populations of leukocytes, (3) release of the NET backbone (DNA) from either nucleus or mitochondria and (4) strategies of pathogens developed to escape from NETs. It turns out that DNA decorated with antimicrobials and proteases is preserved in evolution; not only do all vertebrates (only data on amphibians are missing) release extracellular traps (ETs; Brinkmann et al. 2004; Alghamdi and Foster 2005; Palić et al. 2007; Pijanowski et al. 2013; Reichel et al. 2015) but also invertebrate species (Ng et al. 2013; Homa et al. 2016) and even plants (Wen et al. 2009, 2017) and social amoebae (Zhang et al. 2016) do so. Moreover, although not all cells releasing ETs are leukocytes or leukocyte-like, they all seem to perform a kind of defense function, including root border cells of plants (Hawes et al. 2000) and sentinel cells of the multicellular slug stage of the social amoeba functioning as a primitive innate immune system (Chen et al. 2007). Thus, it is not surprising that, in vertebrates, as depicted in detail in mammals, ET formation is universal among innate immune leukocytes and also characterizes monocytes (Granger et al. 2017), macrophages (Chow et al. 2010; Liu et al. 2014), eosinophils (Yousefi et al. 2008), basophils (Morshed et al. 2014) and mast cells (von Köckritz-Blickwede et al. 2008). Furthermore, the source of DNA can vary since neutrophils and eosinophils not only eject DNA of nuclear but also of mitochondrial origin (mNETs; Yousefi et al. 2008, 2009). The studies on neutrophils revealed that DNA of mNETs indeed contains mitochondrial (e.g., *Cyb*) and not nuclear (e.g., *Gapdh*) genes (Yousefi et al. 2008). Interestingly, mNETs are released by vital neutrophils and they prolong survival of the releasing cells (Yousefi et al. 2009). Finally, different strategies of pathogens to avoid trapping by NETs, or to escape from the released chromatin fibers, have been described. *Streptococcus pneumoniae* and *S.aureus* are good examples of bacteria armed against NETs but fungi (Lee et al. 2015; Rocha et al. 2015; Johnson et al. 2016) and parasites (Guimarães-Costa et al. 2014) have also developed such mechanisms. *S. pneumoniae* possesses the ability to form polysaccharide capsules protecting them from binding to NETs (Wartha et al. 2007) and their endonucleases degrade the network (Beiter et al. 2006). Moreover, *S. pneumoniae* can change the electrical charge of their membrane to positive, by incorporation of D-alanine residues into LTAs (lipoteichoic acids). This strategy protects them against positively-charged residues on NET antimicrobials and proteases preventing the trapping (Beiter et al. 2006). *S. aureus* also releases nucleases but not only to desintegrate NETs (Berends et al. 2010), as they also degrade NET-DNA to intermediate products that are converted to 2′-deoxyadenosine. The latter deoxyribonucleoside induces apoptosis of macrophages that otherwise could phagocytose pathogens immobilized in NETs (Thammavongsa et al. 2013).

Having described the adventages of NET release, one must also acknowledge the side effects of their formation leading to either initiation of bystander damage or even diseases. Numerous studies have reported that uncontrolled and/or excessive release of NETs, as well as their inadequate removal, leads, or at least contributes, to various pathological conditions, including rheumatoid arthritis (RA; Sur Chowdhury et al. 2014; Carmona-Rivera et al. 2017), systemic lupus erythematosus (SLE; Lande et al. 2011; Villanueva et al. 2011), atherosclerosis (Knight et al. 2014; Wang et al. 2017), vasculitis (Kessenbrock et al. 2009; Söderberg and Segelmark 2016), thrombosis (Gould et al. 2014; Martinod and Wagner 2014), sepsis (Kolaczkowska et al. 2015) and cancer (Berger-Achituv et al. 2013; Tohme et al. 2016). SLE and sepsis are representative examples of excessive/inapropiate NET release and inadequate removal, respectively. SLE is manifested by benign skin lesions to life-threatening symptoms resulting from overproduction of autoantibodies and loss of tolerance to their own antigens (Crispín et al. 2010; Dörner et al. 2011). The autoantibodies, anti-neutrophil cytoplasmic antibodies (ANCAs) are directed against PR3, MPO, NE and the anti-nuclear antibodies (ANAs) against DNA and histones, all of which are components of NETs (Fauzi et al. 2004; Yu and Su 2013; Gajic-Veljic et al. 2015). Characteristic for SLE NETs is the presence of LL-37 and human neutrophil peptide (HNP). The DNA/LL-37/HNP complexes activate plasmacytoid dendritic cells (pDCs) resulting in increased production of IFN-α (Lande et al. 2011), which plays a central role in the pathogenesis of SLE by promoting immune system activation that contributes to tissue and organ inflammation and damage (Crow 2014). In addition, NETs of SLE patients are inadequately degraded as they are protected by DNase inhibitors (Hakkim et al. 2010) but also complement C1q bound to NET (Leffler et al. 2012), while LL-37 can protect DNA from degradation (Lande et al. 2011). Of importance, during SLE, numbers of circulating immature neutrophils are elevated (Bennett et al. 2003).

Correspondingly, during sepsis, NETs contribute to bystander damage of endothelium due to activity of histones (Xu et al. 2009; Saffarzadeh et al. 2012; McDonald et al. 2017) and NE (Kolaczkowska et al. 2015) of NETs that are not timely removed. Also, sepsis is characterized by a rapid recruitment to blood of immature neutrophils (Mare et al. 2015) and not fully mature neutrophils are also present in tumors where they display a pro-tumorgenic phenotype (Sagiv et al. 2015). These data suggest that the age of neutrophils might not only impact the phenotype of neutrophils but also their contribution to disease pathology.

## On how NETs are created

Thirteen years into NET research and still we know little about the mechanisms of NET formation, although numerous studies have been published on this topic. Not to underestimate any of the studies, one must keep in mind that, to our estimation, approximately 90% of studies on NETs are performed on isolated neutrophils or tissues collected post-mortem. This does not reflect on a complex in vivo milieu and behavior of neutrophils and other leukocytes in situ, in blood or tissues. However, the main concern is that most of what we know on the mechanisms of NETs come from studies in which PMA was used a sole stimulant. PMA is a syntetic phorbol 12-myristate 13-acetate, a robust activator of two of the three families of protein kinase C (PKC; Liu and Heckman 1998; Neeli and Radic 2013) and, as such, enforces particular signaling pathways. A recent paper re-examing kinetics and signaling pathways of NETs induced by various agents concluded that “PMA stimulation should be regarded as mechanistically distinct from NET formation induced by natural triggers” (van der Linden et al. 2017).

Very early in NET research, dependence on reactive oxygen species (ROS) generated by the NADPH oxidase pathway was reported to be a prerequisite for their formation (Fuchs et al. 2007). The studies were subsequently strongly supported by observation that patients with chronic granulomatous disease (CGD), with impaired NADPH oxidase activity, did not release NETs but that this could be restored by a targeted gene therapy (Bianchi et al. 2009). Subsequently, the Raf-MEK-ERK pathway was identified as being involved in NET formation through activation of NADPH oxidase (Hakkim et al. 2011). But then numerous studies reported ROS-independence of NET formation, which resulted from both in vitro (Gabriel et al. 2010; Byrd et al. 2013; Pijanowski et al. 2013; Mejía et al. 2015) and in vivo studies (Chen et al. 2012; Kolaczkowska et al. 2015; Barth et al. 2016a) utilizing NADPH inhibitors and knockout mice. This discrepancy in the data on ROS involvement in NET release is difficult to explain at this stage. It might be resulting from the experimental milieu or the nature of NET-inducing factors as not all agents activate NADPH oxidase (Farley et al. 2012). The latter study reports on an interesting discrepancy: PMA but not platelet-activating factor (PAF), generated ROS but the NADPH oxidase inhibitor (DPI) reduced NET release by both PMA and PAF. These data indicate that, once again, results from PMA studies should be carefully reviewed unless supported by data from concominant studies applying pathogen- or immune response-related agents to induce NETs. However, most importantly, the study suggests an interesting explanation of ROS involvement in NET formation as DPI also inhibits a range of flavoenzymes including mitochondrial oxidase and nitric oxide synthase (Stuehr et al. 1991; Li and Trush 1998), which could “substitute” for phagosomal ROS. Thus, in some circumstances, NET formation might depend on phagosomal ROS (NADPH-dependent; e.g., Fuchs et al. 2007) but also on mitochondrial ROS (as shown in Lood et al. 2016) or NO (as reported in Patel et al. 2010) or none. It is also of note that the only family of endogenous inhibitors of NETs known to date does not inhibit ROS formation and instead blocks PAD4-dependent citrullination (see “NET formation in neonates”) (Yost et al. 2016).

Another mechanism putatively involved in NET formation is autophagy. This process is critical for the turnover of damaged organelles and proteins during homeostasis but, during infection, plays a role in the killing of phagocytosed pathogens and down-regulation of inflammasome activation (Birmingham et al. 2006; Jabir et al. 2014). The majority of studies showing involvement of autophagy in NET formation applied pharmacological inhibitors of key pathways or molecules involved in this process that however, were also inhibiting ROS (Remijsen et al. 2011; McInturff et al. 2012; Kenno et al. 2016; Ullah et al. 2017). Recently, the involvement of autophagy in NET release was studied in transgenic mice with conditionally deleted *atg5* (its product is critical for autophagosome formation) in either neutrophils or eosinophils (Germic et al. 2017). The study ruled out a role of autophagy in NET formation. A similar controversy concerns the involvement of necroptosis (a programmed necrosis-like cell death), which is well illustrated by two contradictory papers published recently head-to-head (Amini et al. 2016; Desai et al. 2016).

However, there are two enzyme-based mechanisms of NET formation that were confirmed to operate independently of the in vitro or in vivo settings and the inducing agents. These include the involvement of NE and peptidylarginine deiminase 4 (PAD4) (Fig. 1). PAD4 belongs to the group of Ca^2+^-dependent enzymes and is located in the nucleus and granules of neutrophils (Asaga et al. 2001; Nakashima et al. 2002; Kearney et al. 2005). The enzyme is involved in catalyzing the citrullination of histones H2/H3/H4, which is a post-translational modification converting the methylarginine residues to citrulline to form a carbonyl group (Hagiwara et al. 2002; Arita et al. 2006; György et al. 2006). The conversion of positively charged methylarginine to neutral side chains of citrulline affects protein (histone)-DNA stabilization and leads to chromatin decondensation and NET release (Neeli et al. 2008; Wang et al. 2009). Studies on PAD4 knockout mice (PAD4^−/−^) showed impaired ability to form NETs in comparison to WT animals independently of stimuli, be it LPS or ionomycin (Martinod et al. 2013). Similarly, the PAD4 inhibitor (Cl-amidine) also diminishes NET release both in vitro (Li et al. 2010; Kusunoki et al. 2016) and in vivo (Knight et al. 2013, 2014)*.* However, recently, PMA-induced NET formation was reported not to be connected with histone deamination (no citrullinated H3 histones were detected in PMA-induced NETs), which was explained by the fact that PMA activates the PKCα isoform that inhibits PAD4 while it is the PMA-irresponsive PKCζ that activates deamination (Neeli and Radic 2013). Nevertheless, there are also studies reporting deposition of citrullinated histones in PMA-stimulated NETs, although to a lower degree than upon other inducers (Martinod et al. 2016; van der Linden et al. 2017).

Another enzyme required to form NETs is a serine protease: neutrophil elastase. The proposed mechanism of its action is specific degradation of histones that destabilizes chromatin (Papayannopoulos et al. 2010). In addition, blockade of NET formation was also demonstrated in vivo on NE KO mice infected with Gram-negative bacteria (Papayannopoulos et al. 2010; Farley et al. 2012) or Gram-positive bacteria (Kolaczkowska et al. 2015). Also, the use of NE inhibitor resulted in the inhibition of *C. albicans*-induced NET formation (Papayannopoulos et al. 2010). However, Martinod et al. (2016) showed that numerous neutrophils derived from NE^−/−^ mice ejected NETs upon in vitro ionomycin stimulation, while 40% of them did not (Martinod et al. 2016). Interstingly, during mouse sterile thrombosis, only 20% fewer NETs were produced by NE KO neutrophils (Martinod et al. 2016). This indicates that both PAD4 and NE are involved in NET formation but might be more or less redundant depending on the disease state and/or stimuli. For example, during *S. aureus* sepsis, NE^−/−^ neutrophils did not produce NETs while some PAD4^−/−^ PMNs (c. 20%) did (Kolaczkowska et al. 2015), whereas during deep vein thrombosis, 80% of NE^−/−^ neutrophils released NETs (Martinod et al. 2016) but no such structures were cast by the PAD4^−/−^ cells (Martinod et al. 2013). These findings reflect well on the diversity of NET types. The traps seem to vary not only in their appearance, involved molecules and pathways but also in the consequences for the producing cells. The first report on the existence of NETs presented many arguments supporting that the trap-releasing cells remain viable (Brinkmann et al. 2004) but subsequent studies reported on the process being lethal (Fuchs et al. 2007) and eventually a term NETosis was coined (Steinberg and Grinstein 2007). However, Yipp et al. (2012) showed by means of intravital microscopy of *S. aureus*-inflamed skin that anuclear neutrophils that released NETs remain alive and keep moving and phagocytosing (Yipp et al. 2012). This seems more economical and efficient than the beneficial suicide and was detected in the milieu of the live organism. Successively, viable NET-forming neutrophils were also reported in in vitro settings (Yousefi et al. 2009; Pilsczek et al. 2010). Most probably, the two modes represent another set of parallel mechanisms by which NETs are released, either upon cell rupture (Fuchs et al. 2007) or vesicular transport to the cell surface (Pilsczek et al. 2010).

We still do not know how to understand this variety of involved mechanisms and whether reported NETs are always “NETs”, as adequate, multicomponent detection is a key but not a golden standard. This issue is even becoming a topic of open discussions with “healthy critisism” such as the one of Nauseef and Kubes (2016).

## NETs and age of neutrophils

### Immature neutrophils versus mature neutrophils

Neutrophils arise and mature in the bone marrow. The maturation consists of the mitotic stage (myeloblasts, promyelocytes and myelocytes) and postmitotic stage (metamyelocyte, neutrophil band and mature segmented neutrophils) (Borregaard 2010; Amulic et al. 2012; Lahoz-Beneytez et al. 2016). Neutrophil secretion from the bone marrow into circulation is controlled by circadian oscillations (Casanova-Acebes et al. 2013) and depends on the interactions between the CXCL12 chemokine and its CXCR4 receptor (retention of neutrophils in the bone marrow) and the CXCL1 ligand with the CXCR2 receptor (release of neutrophils into blood) (Martin et al. 2003; Eash et al. 2010). In circulation, neutrophil age and human neutrophil half-life is less than 1 day, about 19 h (Lahoz-Beneytez et al. 2016) and about 12 h in mice (Pillay et al. 2010a). Expression of CXCR4 increases on aging cells and causes neutrophils to return to the bone marrow, where they are removed by macrophages (Furze and Rankin 2008; Casanova-Acebes et al. 2013) but the cells can also be removed in the spleen and the liver (Shi et al. 2001; Suratt et al. 2001). In turn, this leads to secretion from the bone marrow of a correspondingly small number of mature but not immature (Bruegel et al. 2004; Nierhaus et al. 2013), neutrophils to the circulation (Semerad et al. 2002). As shown recently, the process is controlled by gut microbiota (Zhang et al. 2015) and most probably also by exosomes whose numbers and content change during aging (Prattichizzo et al. 2017). If during their life neutrophils are recruited to the site of inflammation, their life-span is prolonged and their death by apoptosis is delayed (Simon 2003; Milot and Filep 2011). During inflammation, especially the systemic one, both mature and immature neutrophils are recruited from the bone marrow (Drifte et al. 2013). Interestingly, a recent study showed that the first neutrophils to arrive at the site of inflammation are aged neutrophils and they are followed by nonaged cells (Uhl et al. 2016). The fact that aged cells disappear from circulation, neatly explains why fresh cells are recruited to the blood from the bone marrow in the course of inflammation.

Immature and mature neutrophils differ in their gene expression, the former having higher expression of genes controlling their differentiation and granular protein synthesis, including NE, MPO and BPI, whereas genes controlling chemotaxis or apoptosis are down-regulated in immature neutrophils (Martinelli et al. 2004). Comparison of human immature (bone marrow) and mature (blood) neutrophils in their capacity to produce NETs upon IFN-α/γ priming and following stimulation with complement factor C5, showed that only the mature neutrophils released the traps (Martinelli et al. 2004). Other studies revealed diminished yet detectable NET release by immature neutrophils. In the study by Taneja et al. (2008), circulating neutrophils consisted of c. 35% of immature cells (vs. 5% in healthy volunteers) during sepsis. And similar results were obtained by Pillay et al. (2010b). The immature neutrophils had a lower ratio of phagocytosis and Ca^2+^ signaling (Taneja et al. 2008), antimicrobial recognition and killing and ROS generation (Pillay et al. 2010b). Also, in patients with sterile burn injury, immature neutrophils were numerously present in circulation and these patients had higher levels of circulating free DNA (cfDNA) and citH3, clinical markers of systemic formation of NETs (Hampson et al. 2017). This was especially apparent at times when numbers of immature neutrophils dominated in circulation. However, when neutrophils were isolated from blood and ex vivo-stimulated with PMA, the cells (a mixture of mature and immature neutrophils) of patients with thermal injury released fewer NETs (Hampson et al. 2017).

There is also a report on normal production of NETs by human immature neutrophils present in circulation that comes from studies on bone marrow transplantation (Glenn et al. 2016). Important, although not direct, information on NET production by immature neutrophils comes from studies on diseases during which the undeveloped cells are either present in blood or tissues. One such example is SLE, as lupus patients display a varying degree of neutrophil maturation (Denny et al. 2010; Villanueva et al. 2011). In particular, two neutrophil subpopulations, low-density granulocytes (LDGs) and high-density neutrophils, were identified in the course of the disease. The LDGs do not carry any specific markers identified to date but their nuclear morphology (c. 40% cells have lobular, band or myelocyte-like nuclei vs. c. 60% with segmented nuclei) suggests that many of these cells represent the immature phenotype of neutrophils (Denny et al. 2010). The cells have higher expression of azurophilic granule genes, including those encoding NE and MPO and exhibit increased spontaneous NET production and overall release more traps (Villanueva et al. 2011). A similar subset of neutrophils was also described in the course of psoriasis and psoriatic LDGs also tend to form NETs without any stimulation, in contrast to control or psoriasis mature neutrophils (Lin et al. 2011). Low-density neutrophils, consisting of both immature and mature neutrophils, have also been described in cancer (Sagiv et al. 2015). Unlike high-density neutrophils, the low-density cells have a pro-tumor phenotype (i.e., decreased chemotaxis, phagocytosis and ROS production). The two phenotypes of tumor-associated neutrophils (TANs), i.e., high-density and low-density neutrophils, are also termed N1 and N2, respectively (Fridlender et al. 2009). The N2 phenotype dominates in the presence of TGF-β but is diminished by IFN-β (Fridlender et al. 2009; Andzinski et al. 2016). It was shown that blood neutrophils collected from mice with tumors in which N2 phenotype was suggested to dominate (IFN-β KOs), produced fewer NETs, either spontaneously or upon PMA ex vivo stimulation (Andzinski et al. 2016). These, however, were not TANs and the exact phenotype of circulating neutrophils was not examined, nevertheless immature neutrophils present in a course of disease might not always release spontaneously higher amounts of NETs. In addition, the tumor environment is unique and thus we can speculate that NET release increases anti-tumoral response as NET components might damage tumor cells. But NETs could also function as scaffolds of tumor antigens, facilitating their take-up by DCs and macrophages. On the other hand, NETs can trigger metastasis, e.g., high-mobility group box 1 (HMGB1) released from NETs activates the TLR9-dependent pathway in cancer cells promoting their adhesion, proliferation, migration and invasion (Berger-Achituv et al. 2013; Tohme et al. 2016). Similar results came from a study on immature and mature granulocytes present in leukemic patients (Lukášová et al. 2013). In this study, only data on PMA-induced NETs were reported and acute myeloid leukemia (ALM) granulocytes were shown not to produce the traps as opposed to granulocytes isolated from peripheral blood of healthy donors (Lukášová et al. 2013). The immature cells expressed heterochromatin protein 1 γ (HP1γ) and dimethylated histone H3 at lysine 9 (H3K9me2). The two proteins interact to preserve the spreading of heterochromatin and HP1γ is absent in mature granulocytes. Terminally differentiated mature neutrophils are characterized by a tightly condensed chromatin and gene repression, while immature cells do not (Lukášová et al. 2013). Lukášová et al. (2013) hypothesized that it might be necessary for chromatin to be condensed to facilitate PAD4 action and for this NET formation to be weaker in immature cells.

**Fig. 2:**
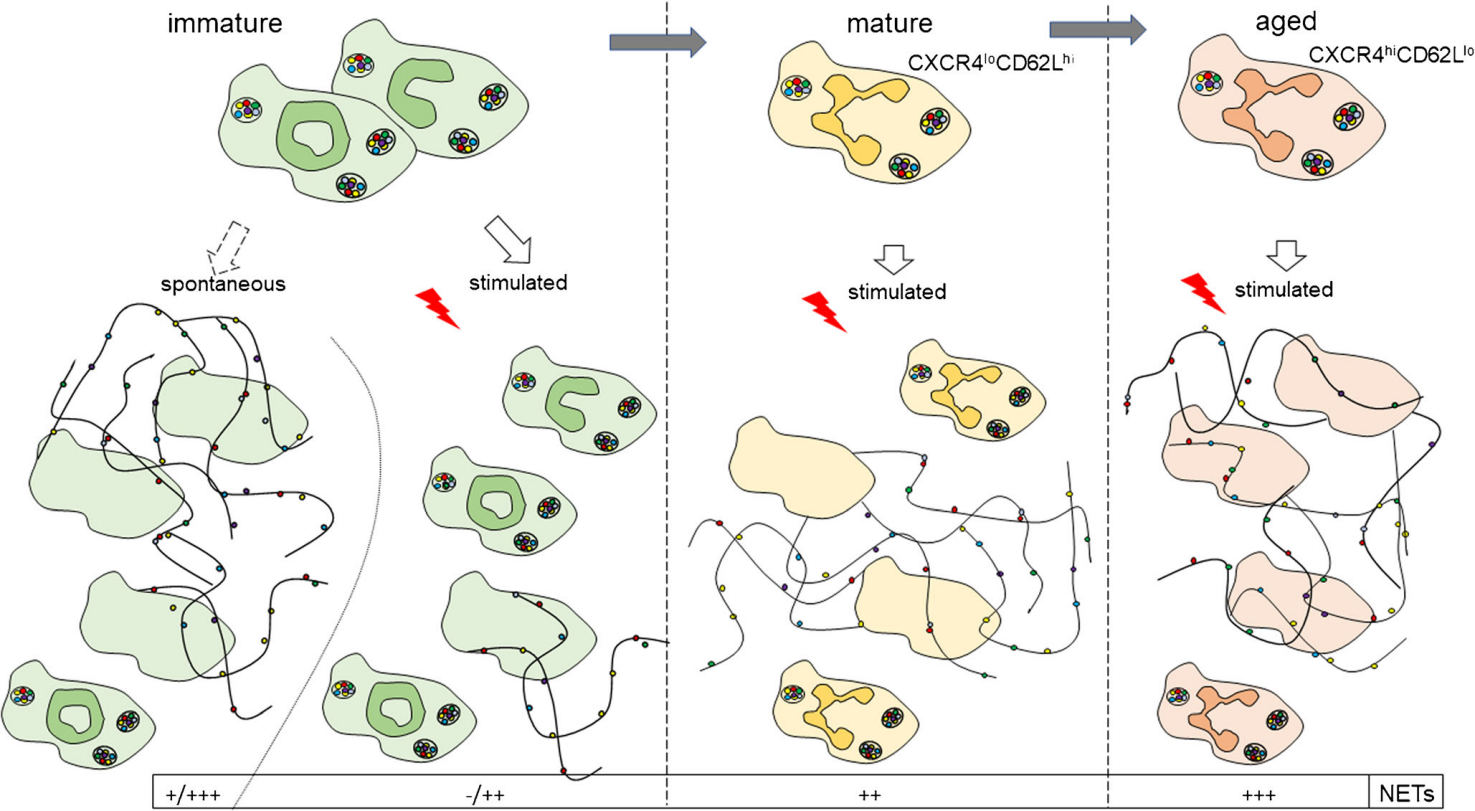
Neurophil maturation- and age-dependent changes in neutrophil extracellular traps (NETs) formation. To strengthen the graphical visualization, potential to form NETs is marked with – and +, where + < ++ < +++; −/+ indicates that, for immature neutrophils stimulated ex vivo, some studies reported a lack of NET formation (−) whereas others reported some NET release although weak (+). Phenotype of mature versus aged neutrophils is defined by high or low expression of CXCR4 and CD62L. Immature neutrophils were mostly defined by their nucleus morphology. Reference data are included and discussed in the main text

One has to bear in mind that the majority of data on NET formation by immature neutrophils come from ill patients (with sepsis, SLE, psoriasis or cancer). Nevertheless, many of them, although not all, report on spontaneous release of the traps by immature neutrophils (if this aspect was studied/reported) and diminished, or at least not futher increased, production of NETs upon stimulation (mostly with PMA) (Fig. 2). In addition, at least one study reported on concomitantly elevated markers of NETs in circulation. Considering all the above data, one might hypothesize that immature neutrophils present in blood tend to spontaneously release NETs, hence the presence of their markers in circulation and thus, when isolated and ex vivo-stimulated to produce the traps, fail to form them. This is either due to an exhausted phenotype of the cells or the fact that all neutrophils with a potential to release NETs have already done so once in vasculature. Especially, it is only about 25% of neutrophils that release NETs (Nauseef and Kubes 2016).

### Aged or senescent neutrophils

Not much is known about NET production by senescent neutrophils. Aging neutrophils up-regulate CXCR4 and progressively lose CD62L (L-selectin) expression that facilitates their re-direction to the bone marrow (Zhang et al. 2015). However, they exhibit enhanced adhesion molecules (e.g., Mac-1, ICAM-1) and TLR4 expression (Zhang et al. 2015), which is in line with their rapid recruitment to the site of inflammation, prior to mature but not aged, neutrophils (Uhl et al. 2016). This aging phenotype is regulated by microbiota and is lost in mice treated with broad-range antibiotics or germ-free animals but restored by application of LPS or fecal transplantation (Zhang et al. 2015). The CD62L^lo^CXCR4^hi^ aged neutrophils are significantly numerous in Selp^−/−^ mice (P-selectin KOs) or anti-P-selectin-treated animals (Zhang et al. 2015; Uhl et al. 2016). When NET production was studied in the latter mice, neutrophils stimulated ex vivo with LPS dramatically increased trap formation. This was further confirmed in an endotoxemic model by intravital imaging of NETs in liver vasculature (Zhang et al. 2015). Therefore, in the case of scenescent neutrophils, the ex vivo and in vivo data clearly correlated, indicating their enhanced capacity to release NETs, which is in line with a pro-inflammatory phenotype of these cells (Fig. 2). However, no data on human scenescent neutrophils are available.

## NETs and age of individuals

Immune system matures during fetal development and then declines as we age. These facts have important impacts on susceptibility to infection and the chances of surviving it. And, as such, it is also important how NET release changes with age. Especially, the world is undergoing a shift in demographics with low birth rates and aging of populations (Boule and Kovacs 2017). Independently of the age of mothers, not only fewer babies are being born but also many of them are born preterm and therefore they are more likely to become ill or die, as preterm infants are more vulnerable to infection (Urquhart et al. 2017). In line with this, the risk of severe sepsis in neonates increases dramatically with decreasing gestational age (Sperandio et al. 2013). On the other hand, the global population is aging and the number of indivuduals older than 65 years will double by 2050 (Boule and Kovacs 2017). Elderly people are more susceptible to infection due to inflamm-aging or immunosenescence, i.e., the age-related dysfunction of the immune system but they also develop chronic inflammatory states (Boe et al. 2017).

### NET formation in neonates

The immune system plays a very important role during pregnancy, with the purpose of protecting the mother and the developing fetus (Mor et al. 2011). Pregnancy is a period that is characterized by modulation of the immune system associated with both the course and stage of pregnancy, as well as the exposure to pathogens. Moreover, the pregnancy is characterized by a pro-inflammatory phase (first trimester), the anti-inflammatory phase (second trimester) and by the end of the pregnancy returns to the pro-inflammatory phase (Mor and Cardenas 2010). Pregnant women have an increase in the total number of leukocytes, which correlates with the course of pregnancy (the highest level is in the third trimester) of which the most abundant cells are circulating neutrophils (Crocker et al. 2000). These neutrophils display a decreased respiratory burst during the second and third trimesters; however, this activity returns to normal within 7 weeks post-partum (Crocker et al. 2000). With respect to NETs, increased levels of cfDNA (nucleosome/MPO complexes) are observed in pregnant women’s serum, compared to nonpregnant women (Sur Chowdhury et al. 2016). Interestingly, the tendency to form such complexes increasingly relates to the duration of pregnancy. Nevertheless, the highest serum cfDNA level is observed in preeclampsia women, as opposed to women with normal pregnancy and nonpregnant women (Lo et al. 1999; Sur Chowdhury et al. 2016). Moreover, the level of both fetal and maternal circulating plasma DNA from preeclampsia women correlates with the degree of disease severity (Zhong et al. 2001).

The fetus, which is located in the uterus, develops its own immune system (Dauby et al. 2012). After birth, both preterm (<37 weeks) and term (37–42 weeks) neonates are characterized by a tolerogenic immune response due to the reduced number of immune cells, including neutrophils or lymphocytes, which increase in the first weeks of life (Walker et al. 2011; Nguyen et al. 2016). In the developing human fetus, a small number of neutrophils begin to appear in the clavicular marrow after 11–12 weeks post-conception with the majority observed after 13–15 weeks (Slayton et al. 1998a, b). However, neutropoiesis starts prior to this in the fetal liver (around week 5; Slayton et al. 1998a) and yolk sac (around week 3; Sperandio et al. 2013). Neutrophils of a mature individual display a capacity to migrate to the site of inflammation and effectively fight pathogens through phagocytosis or degranulation (Kolaczkowska and Kubes 2013). In term neonates, the phagocytosis and degranulation are equally efficient as in adults but not in preterm neonates (Bektas et al. 1990; Nupponen et al. 2002). However, both preterm and term neonates show impaired migration of neutrophils to the inflammatory focus (McEvoy et al. 1996; Nussbaum et al. 2013). Hence, the young organism is not able to defend itself as efficiently as the adult and therefore neonates are highly susceptible to infections, including sepsis, which directly affect increased morbidity and mortality (Gardner 2009; Lawn et al. 2010). Makoni et al. (2016) suggested that impairment of the neonatal neutrophils may be due to the increased number of developmentally immature neutrophils at birth rather than other abnormalities such as the expression of surface adhesion molecules, which is low at birth but increases over time (Carr et al. 1992; Makoni et al. 2016). Another reason could be keeping down immunity to prevent side effects that might result from its overactivity.

**Fig. 3:**
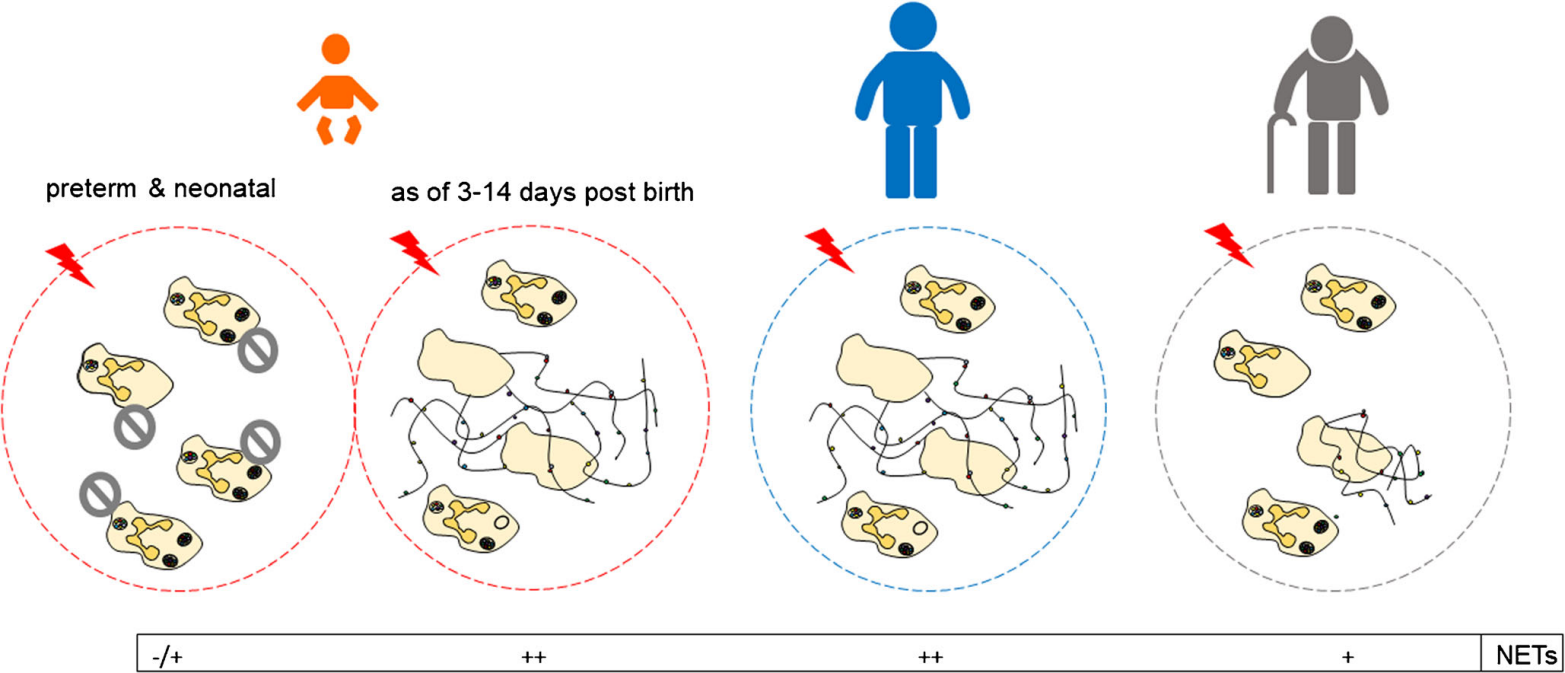
Impact of individual (human) age on neutrophil extracellular trap (NET) release. Graphical representation of neutrophil capacity to produce NETs upon stimulation. In the case of neonates, neutrophil potential to release the traps changes in time. To strengthen the graphical visualization, potential to form NETs is marked with – and + where + < ++ ; −/+ indicates that in the case of newborn infants some studies reported a lack of NET formation (−) whereas others reported some NET release (+). The presence of endogenous NET inhbitors shortly after birth is indicated by* circles with a diagonal line*. Reference data are included and discussed in the main text

Furthermore, the formation of NETs in preterm or term infants/neonates has been reported to be weaker (Fig. 3). Neutrophils isolated from infants/neonates displayed impaired NET production after stimulation with LPS, PAF and fMLP, in contrast to neutrophils collected from adult individuals (Yost et al. 2009; Lipp et al. 2017). This was despite the presence of functional receptors that recognize these molecules and uncompromised phagocytosis. Nevertheless, when bacteria (*E. coli*, *S. aureus*) or PMA were used to induce NETs, neonatal neutrophils did not form NETs (Yost et al. 2009). On the other hand, Lipp et al. (2017) reported that the cells of term infants release some NETs in response to PMA and those of preterm babies release significantly fewer of these structures. Importantly, the defect of NET formation by neutrophils of preterm and term neonates was not rescued by the ROS donor (glucose oxidase) (Yost et al. 2009). Also, the study by Byrd et al. (2016) showed that NETs induced by neonates in response to a combination of fibronectin (Fn) with purified fungal β-glucan or Fn with *C. albicans* hyphae are ROS-independent, although in this case, NETs were formed normally. Thus, neonate neutrophils seem to be sensitive to fungal stimulation but not necessarily the bacterial components (Byrd et al. 2016). However, in contrast to Lipp et al. (2017), Marcos et al. (2009) showed that neonatal neutrophils can cast NETs upon LPS (as well as other numerous TLR agonists) although at first the signal is weaker (Marcos et al. 2009). Direct comparison of the two studies indicates that neonate neutrophils release NETs but they require a longer time for their maximal formation. In fact, further studies revealed that even the most prematurely born infants gain the capacity to release NET by day 3 post-birth and maximal capacity to cast NETs is achieved between day 3 and 14 of life (Yost et al. 2016). This characteristic seems also to be present in other mammals, as the same phenomenon was observed in pigs (Nguyen et al. 2016). Also, neutrophils of 21-day-old mice produced fewer NETs than the cells of 60-day-old animals (Barth et al. 2016b).

In the search for mechanisms of impaired/delayed NET formation by neonates, a family of endogenous inhibitors of NETs was discovered (Yost et al. 2016). The family, called nNIF-related peptides (NRPs), after the first identified peptide (NET-inhibitory factor, nNIF), also consists of cancer-associated SCM recognition, immune defense suppression and serine protease protection peptide (CRISPP) and a 44–amino acid carboxy terminus cleavage fragment of A1AT (α1-antitrypsin), A1ATm358 (Yost et al. 2016). The levels of inhibitors rapidly decrease in the circulation of the infant after delivery. This might explain why, in some studies, differences in NET formation were reported between preterm and term infants. The inhibitors were detected in different tissues/body fluids - umbilical blood (nNIF), placenta (A1ATm358), plasma (CRISPP-related peptides) - underlinging their importance. They also inhibited NET formation induced by bacteria (*S. aureus*), damage-associated molecular pattern (DAMP; heme) and PMA (Yost et al. 2016) but did not destroy them. A mechanism of their action is also very intriguing, as NRPs do not affect ROS production nor NE activity (although, after entering the cell, they localize in its close proximity) but inhibit PAD4 and histone citrullination. Importantly from a therapeutical point of view, the injection of either nNIF or CRISPP into adult mice infected with *E. coli* or LPS prevented formation of NETs and decreased mortality (Yost et al. 2016).

Why would such inhibitors function only in fetuses/neonates? nNIF levels were negligible in healthy adults and undetectable in the plasma of adult individuals with chronic inflammatory disorders (Yost et al. 2016). It is possibly because, during pregnancy, NET-inducing stimuli are present/generated at the maternal fetal interface (Gupta et al. 2005; Marder et al. 2016; Mizugishi and Yamashita 2017) and thus excessive formatiom of the traps could cause inflammatory pathology in the fetomaternal environment. But then, shortly after birth, the inhbitors are degraded or neutralized by unknown means. Intriguingly, the latter correlates with the time when resident microbiota inhabits the human infant and the microbiota was indeed shown to regulate granulocytosis and host resistance to sepsis in the neonate (Deshmukh et al. 2014). The impact of microbiota on the life-span and functioning of neutrophils in adulthood has just been established (see “NETs and age of neutrophils”).

### NET release by elderly individuals

An aging organism, like the newborn, is susceptible to a variety of inflammatory pathogenesis, leading to increased morbidity, which is due to impaired immune function (Collerton et al. 2012; Tseng et al. 2012; Boe et al. 2017). Therefore, the term immunosenescence has been introduced. Immunsenescence, or inflamm-aging, is associated with low-grade, chronic, pro-inflammatory status, resulting from an imbalance between pro-inflammatory agents and anti-inflammatory factors (Franceschi et al. 2007; Collerton et al. 2012). It is characterized by elevated levels of pro-inflammatory cytokines, including IL-6 and TNF-α, in physiological conditions (Bruunsgaard et al. 2000; Krabbe et al. 2004; Ferrucci et al. 2005). One hypothesis says it is because of the constant immune challenges over the lifetime leading to a higher basal activation state of cells of the innate immune system (Fulop et al. 2017). In addition, a recent study reports that these age-associated changes depend on the microbiota (Thevaranjan et al. 2017). On the other hand, the elderly have a weaker response to vaccination (Goodwin et al. 2006; Sasaki et al. 2011), which might result from an impaired ability to present antigens to T cells, the latter leading to a dysfunctional immune response (De Martinis et al. 2004; Plowden et al. 2004; Wong and Goldstein 2013).

Neutrophils of elderly individuals are characterized by impaired bactericidal activity (Wenisch et al. 2000), chemotaxis (Fulop et al. 2004), phagocytosis (Butcher et al. 2001; Simell et al. 2011) and decreased ability to perform a respiratory burst (Wenisch et al. 2000). However, some parameters are either preserved (chemokinesis) or up-regulated (degranulation) (Sapey et al. 2014). The changes are believed to reflect on the behavior of the cells in aged individuals. They perform aberrant migration (altered chemotaxis/chemokinesis ratio) so they can spread more efficiently than those from younger individuals and, because they release more protease (as shown for NE; Sapey et al. 2014), possibly to facilitate migration through the ECM, more collateral damage can occur.

Interestingly, high levels of NE, along with eleventated pro-inflammatory cytokine levels, are also characteristic for the low-grade inflammatory state accompanying obesity (Talukdar et al. 2012). In fact, it is recognized now that such an inflammatory state connects aging, metabolic syndrome and cardiovascular disease (Guarner and Rubio-Ruiz 2015).

In physiological conditions, numbers of neutrophils in the bone marrow are similar between old and young mice. However, during inflammatory conditions, such as sepsis induced by cecal ligation and puncture (CLP), fewer neutrophils are observed in the peritoneal lavage in old versus young mice (Xu et al. 2017). Thus, while in healthy aged organisms, the pro-inflammatory state is apparent, in the course of inflammation, the immune response seems to be dimmed. Although not many studies have been undertaken on NET formation by elderly individuals, they all consistently reported weaker production of the traps, in line with the data on other neutrophil activities (Fig. 3). It was observed when the cells were first primed with TNF-α and then activated to form NETs with LPS or IL-8 (Hazeldine et al. 2014), stimulated with Pam3CSK4, a TLR2 ligand (Xu et al. 2017), *S. aureus* (Tseng et al. 2012) or mitochondrial DNA, a DAMP (Itagaki et al. 2015). Notably, expression of nucleases by *S. aureus* (vs. the nuclease null strains) led to increased bacterial dissemination in young but not old mice, suggesting that defective NET formation in elderly mice permitted both nuclease and non-nuclease expressing *S. aureus* to disseminate, altogether leading to more invasive *S. aureus* infection (Tseng et al. 2012). Interestingly, neutrophils isolated from elderly periodontitis patients also released fewer NETs than the young ones but this was not observed in the case of healthy age-matched controls (Hazeldine et al. 2014). In the studies applying TLR2 and TLR4 ligands, neutrophils collected from elderly people had normal expression of respective receptors required for the cell activation but dimished ROS production (Hazeldine et al. 2014; Xu et al. 2017). And thus the latter was proposed as a mechanism of the lower NET release. However, Hazeldine et al. (2014) as well as Tortorella et al. (2004) showed that there is no impairment in p38 mitogen-activated protein kinase (p38 MAPK) activity, the signaling cascade activated by ROS, in TNF-α-primed neutrophils in both the elderly and younger individuals. Furthermore, PMA, a strong chemical inducer of ROS, induced similar quantities of NETs in both age groups (Tortorella et al. 2004; Hazeldine et al. 2014).

Another possible mechanism leading to dimished NET formation in aged individuals is impaired autophagy. Although involvement of autophagy in NET formation is controversial (see “On how NETs are created”), its impairment was suggested to be co-responsible, along with ROS, for a weak NET release by neutrophils of elderly individuals (Xu et al. 2017). In particular, a defect of Atg5, involved in autphagosome formation, was pointed out to contribute to dimmed NET release. And instead of forming NETs, neutrophils were undergoing apoptosis (Xu et al. 2017) .

There is one report on an increased capacity of neutrophils from aged individuals to produce NETs. This observation comes from studies on aortic lesions in atherosclerotic mice and is strengthened by data from isolated neutrophils activated to produce NETs with 7-ketocholesterol, an athero-relevant stimulus, the most abundant oxysterol in human (Wang et al. 2017). Such an effect resulted from increased mitochondrial oxidative stress, thus mitochondrial (mitoOS) and not cytosolic, ROS generation. The former being indeed associated with atherosclerosis during aging (Vendrov et al. 2015). Considering that numbers of inflammatory neutrophils were the same in aged and young mice, the young animals had smaller lesions and their NET formation was mitoOS-dependent, indicating intrinsic changes in neutrophils of aged subjects. This experimental setting differs from the other studies on NET formation by neutrophils of elderly individuals in clear requirement of mitochondrial ROS and not NADPH oxidase-dependent.

As for what we know to date, neutrophils of elderly subjects in general cast fewer NETs (Fig. 3). No data indicate so far that this is because of active inhibiton of their formation as in neonates but rather it results from dysregulated activity of neutrophils. It is tempting to speculate that one of the mechanisms involved might be connected to the increased release of NE via degranulation, as this enzyme is critical for NET formation. For these NETs that require NADPH oxidase-dependent ROS, a diminished respiratory burst by neutrophils of elderly subjects can provide an additional explanation. However, the observation that increased mitochondrial ROS can in fact increase NET formation by neutrophils of aged individuals suggests that the cells do not lose the capacity to release NETs per se and that this is rather due to upstream dysfunctional pathways.

## Conclusions

The phenotype of any given cell reflects either its maturation state or the impact of extrinsic factors and manifests itself by changes in cell morphology, expression pattern of intracellular and extracellular molecules but, foremost, its (altered) functioning. This is also true for neutrophils and their capacity to induce NETs. As, nowadays, NETs are the focus of biomedical research, mostly due to the side effects of their formation, a search for their inhibitors or removing agents dominates the field. Owing to studies on neonate neutrophils is the discovery of endogenous NET inhibitors. This is especially promising in the light of finding that immature neutrophils, which are more abundant in numerous diseases in which NETs play a pivotal role, release the traps spontaneously. Obviously, the cells do not behave uniformly in all conditions and studies on NETs are also technically challenging as they mostly rely on either detection of singular NET components in body fluids or ex vivo stimulation of isolated neutrophils. Although, in the case of mice studies, these limitations can be overcome with intravital microscopy, detecting the traps directly in blood vessels or tissues of live animals, this technique cannot be applied to human studies. And NET inhibition can also be detrimental. For instance, at early stages of sepsis, the structures help to contain dissemination of infection and it is at later time points that their persistent presence causes collateral damage. Thus, NET inhibition or removal should also be timely adjusted, which, however, is difficult to control. Now, a new factor has to be taken into consideration when it comes to the control of NET formation and its consequences, namely the presence of neutrophils of certain ages (immature–mature–senescent) as well as the age of the individuals.
